# Malaria in 2022: Increasing challenges, cautious optimism

**DOI:** 10.1038/s41467-022-30133-w

**Published:** 2022-05-13

**Authors:** Prasanna Jagannathan, Abel Kakuru

**Affiliations:** 1 Department of Medicine, Stanford University, Stanford, CA United States; 2 Department of Microbiology and Immunology, Stanford University, Stanford, CA United States; 3 Infectious Diseases Research Collaboration, Kampala, Uganda

**Keywords:** Antiparasitic agents, Vaccines, Parasitology

## Abstract

Malaria cases and deaths remain unacceptably high and are resurgent in several settings, though recent developments inspire optimism. This includes the approval of the world’s first malaria vaccine and results from novel vaccine candidates and trials testing innovative combinatorial interventions.

Despite gains over the first 15 years of this millennium, malaria control has stagnated in the last several years, with resurgence and rising morbidity in several highly endemic countries exacerbated by service disruptions due to the COVID-19 pandemic^1^. In 2020, malaria was estimated to have resulted in 627,000 deaths and 241 million cases, with 77% of deaths in children <5 years of age^1^. Overall, 90% of malaria cases and deaths are reported in Africa, and six countries—Nigeria, DRC, Uganda, Mozambique, Angola, and Burkina Faso—account for 55% of all cases globally.

The main interventions used for prevention of malaria include vector control with long lasting insecticidal bednets (LLINs) and indoor residual spraying of insecticides (IRS). However, *Anopheles* vector resistance to pyrethroids, the main insecticide used in LLINs, has become widespread, and insecticide resistance also increasingly threatens the utility of IRS. In addition to vector controls, prompt treatment of malaria with artemisinin-based combination therapy (ACTs) is recommended in all settings where falciparum malaria is endemic. ACTs have played a crucial role in controlling malaria over the past 20 years^2^, with artemether-lumefantrine being the most widely used ACT in Africa. However, artemisinin-resistant *Plasmodium falciparum* parasites have spread in Southeast Asia^3^, resulting in reduced treatment efficacy of some ACTs^4^. More alarmingly, recent reports from Rwanda^5,6^ and Northern Uganda^7,8^ suggest the emergence of artemisinin-resistant parasites in Africa. Loss of artemesinin activity would threaten the activity of partner drugs such as lumefantrine; loss of both components of ACTs could have devastating consequences across the continent^9^.

To combat the emergence of artemisinin-resistant parasites, identification of novel therapeutic approaches has become critically important. Although new antimalarial drugs are being identified, they are still in various stages of clinical development (www.mmv.org). One such drug, KAF-156, was found to be active against artemisinin-resistant parasites in a small trial of adults^10^, and is currently being tested in Phase 2 trials in children when given with the partner drug lumefantrine (NCT 04546633). Another emerging therapeutic strategy is the use of artemisinins along with two long-acting partner drugs instead of one, similar to the therapeutic approach to HIV and tuberculosis (e.g., triple ACTs.). Triple ACTs have been found to be effective in clinical trials conducted in the setting of artemisinin-resistant parasites^11,12^, and may be useful as a “stop-gap” therapy for drug-resistant malaria until new antimalarials become available, or to prevent and/or delay the development of resistance to antimalarials in settings where resistance has not yet emerged^13^. Given potential safety and tolerability concerns, questions remain about which agents to use, and how and when to deploy such a strategy.

One of the most elusive interventions to aid malaria control has been an effective malaria vaccine that can prevent severe malaria and deaths. The WHO and partners set a strategic goal of achieving a malaria vaccine with >75% efficacy by 2030^14^. However, this goal has been a major challenge, and very few candidate vaccines have demonstrated significant efficacy^15^. The malaria vaccine RTS,S/AS01 is the only vaccine tested to reach Phase 3 trials with reproducible efficacy in different populations. This recombinant protein vaccine targets the circumsporozoite protein (CSP) of *P. falciparum*, which is expressed at the pre-erythrocytic stage of infection. Although RTS,S has been found to be efficacious, overall efficacy in the Phase 3 trial was low, with 36% protective efficacy against clinical malaria, and 32% against severe malaria, in the 4 years after vaccination among children who began the vaccination series between 5 and 17 months of age and received a booster 21 months later^16^. RTS,S efficacy was highest (~60–70%) in the first 6 months following vaccination, but rapidly decayed, and was limited or non-significant by 18 months^17^. This waning of vaccine efficacy was broadly mirrored by a decline in antibody responses against CSP^18^, although definitive correlates of protection remain unclear. Identification of correlates and mechanisms that contribute to malaria vaccine performance in endemic settings remains an active area of research.

Given results from the Phase 3 trial, the WHO launched pilot implementation studies of RTS,S in Malawi, Kenya and Ghana beginning in 2019^19^. These implementation studies showed that delivery of the vaccine was feasible, with high uptake of the vaccine, confirming demand. Importantly, these studies also reaffirmed vaccine safety and efficacy, observing that vaccination was associated with a 30% reduction in severe malaria^1^. Given these results, in October of 2021, after 30 years of development, the WHO-approved RTS,S for use in children living in regions with moderate to high transmission of malaria caused by *P. falciparum*.

Although RTS,S is now WHO-approved, its availability will be limited in the short term. GlaxoSmithKline (GSK), which produces the vaccine, is committed to donate up to 10 million vaccine doses to the pilot implementation regions of Ghana, Kenya, and Malawi through 2023, and to supply up to 15 million doses of vaccine per year to the end of 2028 if it is recommended for wider use, pending financing. However, this represents only ~10–15% of the annual doses required if provided to all children living in highly endemic settings^20^. GSK has committed to transfer the technology to manufacture RTS,S to Bharat Biotech (BBIL), and, by 2029, BBIL is expected to be the sole supplier of the vaccine, with increased production capacity expected. Given the relatively low efficacy of RTS,S, and its limited short-term availability, new vaccines are needed to reach the WHO target of malaria vaccines with >75% efficacy by 2030. One such candidate is R21, another CSP-based subunit vaccine with a similar construct to RTS,S but with more CSP antigen in the virus-like particle. In a phase 2 trial, R21 was recently shown to have 71–77% protective efficacy against a first episode of clinical malaria in the year following vaccination among children living in an area with seasonal malaria transmission^21^. However, this study only reported protection across one malaria transmission season; the durability of this protection, and whether R21 would be efficacious in areas with year-round malaria transmission, remains unclear. Phase 3 trials in 4 countries, with longer follow-up, are underway. In addition, several other vaccines for both *P. falciparum* and *Plasmodium vivax* are under development, targeting each of the life cycle stages of Plasmodium, including sporozoite/pre-erythrocytic, asexual/erythrocytic, and sexual/mosquito^22^.

Another promising intervention for malaria control is intermittent preventive therapy (IPT)—the provision of full treatment doses of antimalarial drugs to at risk populations to clear existing infections and prevent new infections. IPT with sulfadoxine-pyrimethamine (SP) given at the time of routine vaccination in infants (IPTi) has been shown to be safe and modestly effective against malaria in the first year of life^23^, but is only recommended in areas with low levels of SP resistance. Uptake of IPTi has therefore been very low, with only one country (Sierra Leone) recently adopting this strategy. Seasonal malaria chemoprevention (SMC) using monthly SP plus amodiaquine during the transmission season is a proven strategy to decrease morbidity and mortality in young children^24^, and is currently deployed in parts of West and Central Africa where annual malaria transmission is confined to a few months. However, neither IPTi nor SMC are recommended in areas with high level SP resistance and/or year-round malaria transmission as in much of Central and East Africa^25^. In these settings, the ACT dihydroartemisinin-piperaquine (DP) has emerged as an excellent candidate for use as IPT in children^26^, including as perennial malaria chemoprevention in areas with year-round malaria transmission^27^. IPT during pregnancy (IPTp) with DP has also been shown to be more effective than IPTp with SP for prevention of malaria in pregnancy in areas with high level SP resistance^28,29^, although IPTp with SP may result in improved birth outcomes independent of SP’s antimalarial activity^30^. As above, there are important concerns about selection of drug-resistance through IPT, though modeling suggests that this could be limited via prevention of infections and/or optimization of target drug concentrations^31^. IPT studies should therefore be accompanied by close monitoring for emergence of genotyping and phenotypic evidence of antimalarial drug resistance. An added concern is that preventing malaria in children may delay acquisition of antimalarial immunity, increasing the risk of malaria after IPT has stopped (rebound malaria). Though some studies have reported rebound following cessation of IPT^32,33^, other studies have reported either no increase^23^ or evidence of sustained protection^27,34^ following cessation. How IPT impacts the acquisition of immunity to malaria remains an important area of investigation.

Finally, exciting new data suggest that combinations of malaria control interventions might be more efficacious than individual interventions. Vaccination of malaria-naïve adults with *P. falciparum* sporozoites under prophylactic cover with either chloroquine or pyrimethamine induced durable sterile protection against controlled challenge with either a homologous or heterologous *P. falciparum* strain^35^. A follow-up study conducted in malaria-exposed Malian adults has recently been completed (NCT03952650), with results eagerly anticipated. However, studies of this strategy in malaria-exposed children will be needed, given prior vaccination studies showing limited efficacy in children despite higher vaccine efficacy in adults^36^. In another recent randomized controlled trial conducted in West Africa, investigators found that the combination of seasonal malaria chemoprevention (SMC) in children with amodiaquine + sulfadoxine-pyrimethamine (AQ + SP) along with RTS,S vaccination was superior to either intervention alone^37^. This promising dual intervention deserves additional study in settings where malaria transmission is seasonal. However, in settings with year-round malaria transmission and/or high SP resistance, alternative IPT + vaccine regimens require urgent evaluation.

In conclusion, despite earlier gains, malaria cases and deaths remain unacceptably high and are resurgent in several settings, and our ability to prevent and control malaria with current tools is challenged by the specter of insecticide-resistant vectors and drug-resistant Plasmodium parasites. Clearly, renewed focus—and new interventions—are needed to achieve the goals highlighted by the WHO “high burden to high impact” campaign to reduce cases and deaths in countries hardest hit by malaria. There are reasons for cautious optimism, including approval of the world’s first malaria vaccine and results from novel vaccine candidates and trials testing innovative combinatorial interventions. However, critical research gaps remain, and there is an urgent need to prioritize and fund development of novel therapeutic, prophylactic, and vaccine strategies against malaria.

## Reporting summary

Further information on research design is available in the Nature Research Reporting Summary linked to this article.

## Supplementary information

Reporting summary
